# Graphene perfect absorber with loss adaptive *Q*-factor control function enabled by quasi-bound states in the continuum

**DOI:** 10.1038/s41598-021-02318-8

**Published:** 2021-11-24

**Authors:** Sangjun Lee, Joohyung Song, Sangin Kim

**Affiliations:** Department of Electrical and Computer Engineering, Ajou University, Suwon, 16499 Korea

**Keywords:** Optics and photonics, Electronics, photonics and device physics

## Abstract

Numerous device structures have been proposed for perfect absorption in monolayer graphene under single-sided illumination, all of which requires the critical coupling condition, i.e., the balance between the loss of graphene and the leakage rate of the device. However, due to the difficulty of the precise control of the quality of synthesized graphene and unwanted doping in graphene transferred to the substrate, the loss of graphene is rather unpredictable, so that the perfect absorption is quite difficult to achieve in practice. To solve this problem, we designed a novel perfect absorber structure with a loss adaptive leakage rate control function enabled by the quasi-bound states in the continuum (BIC) and numerically demonstrated its performance. Our designed device is based on a slab-waveguide grating supporting both the quasi-BIC and the guided-mode resonance (GMR); the quasi-BIC with an adjustable leakage rate controlled by an incident angle is responsible for absorption, while the GMR works as an internal mirror. Since the proposed device scheme can have an arbitrarily small leakage rate, it can be used to implement a perfect absorber for any kind of ultrathin absorbing media. Due to the simple structure avoiding an external reflector, the device is easy to fabricate.

## Introduction

Graphene has attracted strong interests in developing high-speed photodetectors due to its high carrier mobility^1–4^. Undoped monolayer graphene exhibits uniform light absorption efficiency of ~ 2.3% over a wideband wavelength range from visible to terahertz. Considering its atomically ultrathin thickness of ~ 0.34 nm, the absorption efficiency is relatively high value. However, for practical high-performance photodetectors, the absorption should be greatly enhanced by applying various resonant structures such as gratings or photonic crystals^5–8^, prism couplers^9,10^, and Fabry–Perot cavities^11,12^. From a standpoint of absorption efficiency enhancement, perfect absorption under single-sided illumination is an ultimate solution. Over the past decade, three types of perfect absorber schemes have been proposed: a single-mode/mirror scheme^5–16^, a dual-mode (or degenerate critical coupling) scheme^17,18^, and a triple-mode scheme^19^. The single-mode/mirror scheme requires a rather complicate fabrication process due to Bragg reflector or suffers from unwanted loss due to metal reflector. Although the dual-mode scheme avoids the use of mirror, it is prohibitively restrictive to simultaneously satisfy the frequency degeneracy and the critical coupling conditions of dual modes. The triple-mode scheme relieves design complexity compared to the dual-mode scheme but requires somewhat complicate fabrication process due to an added slab structure. Recently, our group proposed another mirror-less perfect absorber scheme based on an asymmetric single resonator such as a slab-waveguide grating (SWG) by adopting a one-port mimicking concept^20^. In this scheme, two indirectly coupled degenerate guided-mode resonance (GMR) modes are used to achieve a virtual one-port system, in which only one mode experiences loss and the other functions as an internal reflector in conjunction with the Fabry–Perot (F-P) like background scattering. This scheme does not require any structural symmetry, and its performance shows greatly enhanced fabrication error tolerance in comparison to the previously proposed dual-mode scheme of ref.^17^.

In the perfect absorber schemes, the critical coupling condition, i.e., balance between loss and leakage (or decay) rates is a common and essential requirement. The leakage rates of the relevant resonant modes in all the forementioned perfect absorber schemes are determined by structural parameters and thus, can hardly be adjusted after fabrication. On the other hand, the loss rate of graphene is somewhat unpredictable since the quality and the thickness of synthesized graphene is quite difficult to control precisely^10^. Besides, the substrate can cause unwanted doping of graphene, which also changes the loss rate. So, the critical coupling condition for perfect absorption is quite difficult to satisfy in practice. This is the fundamental problem of all the previously proposed perfect absorber schemes. In this work, to solve this problem, we newly propose a perfect absorber scheme with a controllable leakage rate based on a quasi-bound states in the continuum (BIC).

The BIC is a special eigenstate that remains localized and has an infinite quality factor (*Q*-factor) even though it exists within the light cone of the surrounding medium^18,21–36^. In practice, the BIC can be realized as a quasi-BIC whose finite *Q*-factor is adjusted by breaking structural symmetries^18,24–32^ or through parameter tuning^33–36^. Recently, quasi-BICs in various periodic structures (such as gratings or photonic crystals^23–28,33–35^, and metasurfaces^18,29–32^) or an isolated nanoparticle^36^ have attracted substantial attention as a platform for enhancement of light-matter interaction due to their ultrahigh *Q*-factor.

An interesting feature of the quasi-BICs in the periodic structures formed by breaking structural symmetries with oblique incidence of light is that their *Q*-factors are dependent on the incident angle^24–28^. Based on the controllable *Q*-factor of the quasi-BIC, we devised the practical perfect absorber scheme in which its leakage rate is adapted to the loss rate by a proper choice of the incidence angle. The basic concept of our proposed perfect absorber is the same as the asymmetric resonator-based one-port mimicking scheme^20^. Here, the quasi-BIC is adopted in the SWG as the lossy resonant mode and its resonance should be tuned to the GMR and the background scattering which forms an internal reflector function. In addition to the adaptive leakage rate, another outstanding feature of the proposed perfect absorber is considerably simple fabrication since absorbing medium (graphene) is placed on the ridge side of the SWG. This is because the quasi-BIC is mostly confined in the ridge region. In the designed device, for small value of asymmetry parameters, which is determined by an incidence angle, quadratic dependence of the *Q*-factor on the asymmetry parameter is confirmed. To investigate the reflection and absorption spectra, and field profiles of various resonant modes in the proposed absorber, we used the rigorous coupled wave analysis (RCWA) method^37^. The incidence angle dependence of quasi-BIC is also analyzed by using the coupled mode theory (CMT)^5,6,38^.

## Results & discussion

**Figure 1:**
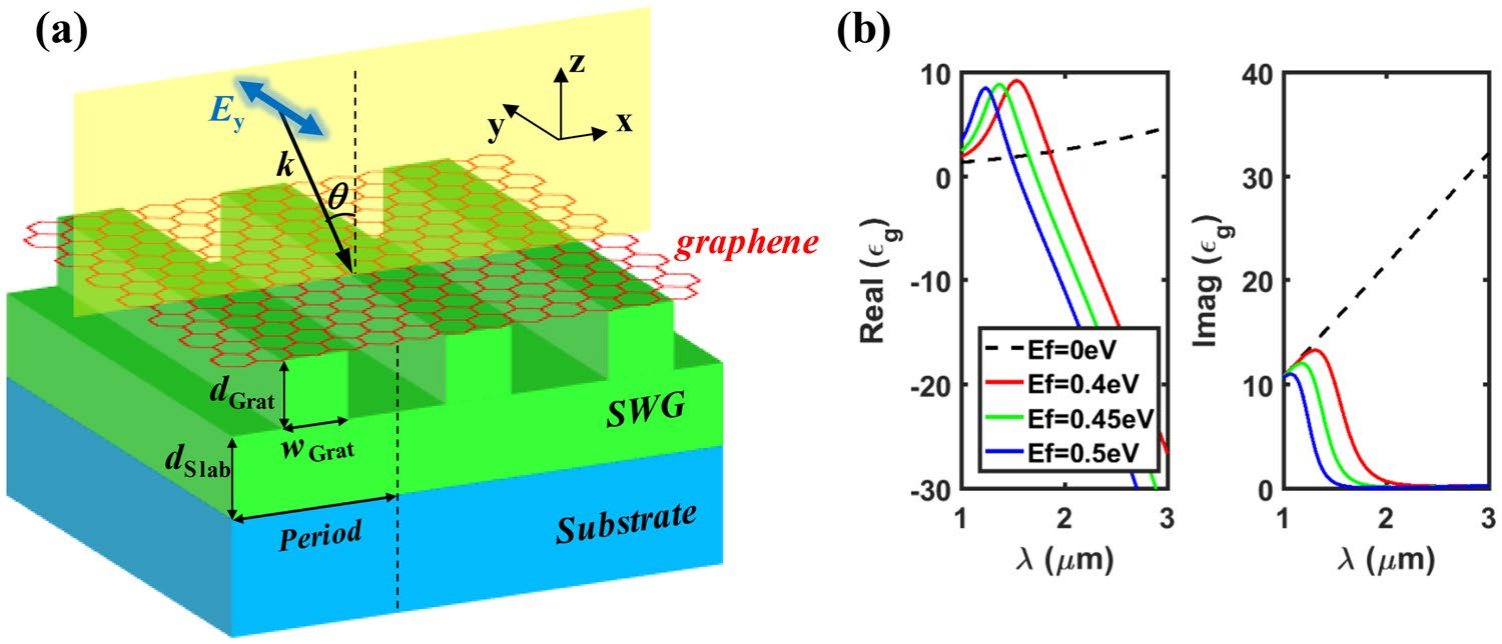
(**a**) Schematic of the proposed perfect absorber consisting of monolayer graphene placed on a slab-waveguide grating (SWG), where *n*_SWG_ = 3.40, *n*_Sub_ = 1.45. *FF* is defined as *w*_Grat_/*Period*. The red thin layer indicates monolayer graphene of *t*_*G*_ = 0.34 nm thickness as an absorbing medium. (**b**) Real and Imaginary part of permittivity ($\varepsilon$_g_) of graphene for different Fermi-levels (*E*_f_ = 0, 0.4, 0.45, and 0.5 eV) over the wavelength range of $\lambda$ = 1–3 $\upmu$m. The optimal (*A* > 99.99%) design parameters for *E*_f_ = 0: *Period* = 0.85 $\upmu$m, *d*_Grat_ = 0.367 $\upmu$m, *d*_Slab_ = 0.052 $\upmu$m, *FF* = 0.55, and $\theta$ = 3.72 deg at $\lambda$ = 1.5472 $\upmu$m.

The proposed absorber (Fig. 1a) consists of a one-dimensional (1-D) SWG of Si stacked on a low-index substrate (SiO_2_), where the ridge (grating) and the sublayer (slab) possess the same index, and monolayer graphene placed just above the SWG. Refractive indices of Si and SiO_2_ are 3.40 and 1.45, respectively. For the permittivity of graphene, Kubo formulation^19,39^ was used with parameters of a graphene thickness of 0.34 nm, Fermi velocity of 10^6^ m/s, and mobility of 0.5 m^2^/Vs. Figure 1b shows real and imaginary parts of the permittivity ($\varepsilon$_g_) of graphene for different Fermi-levels (*E*_f_ = 0, 0.4, 0.45, and 0.5 eV) over the wavelength range of $\lambda$ = 1 ~ 3 $\upmu$m. We consider a transverse electric (TE, or *s*-polarized) wave obliquely incident from the air with an incidence angle of $\theta$, whose electric field is perpendicular to the incidence plane. The fill factor (*FF*) is defined as *w*_Grat_/*Period*. The optimal design parameters are as follows: *Period* = 0.85 $\upmu$m, *d*_Grat_ = 0.367 $\upmu$m, *d*_Slab_ = 0.052 $\upmu$m, *FF* = 0.55, and $\theta$ = 3.72 deg at $\lambda$ = 1.5472 $\upmu$m, which are designed to obtain perfect absorption (*A* > 99.99%) in undoped (*E*_f_ = 0) monolayer graphene at $\lambda$ ~ 1.55$\upmu$m.

**Figure 2:**
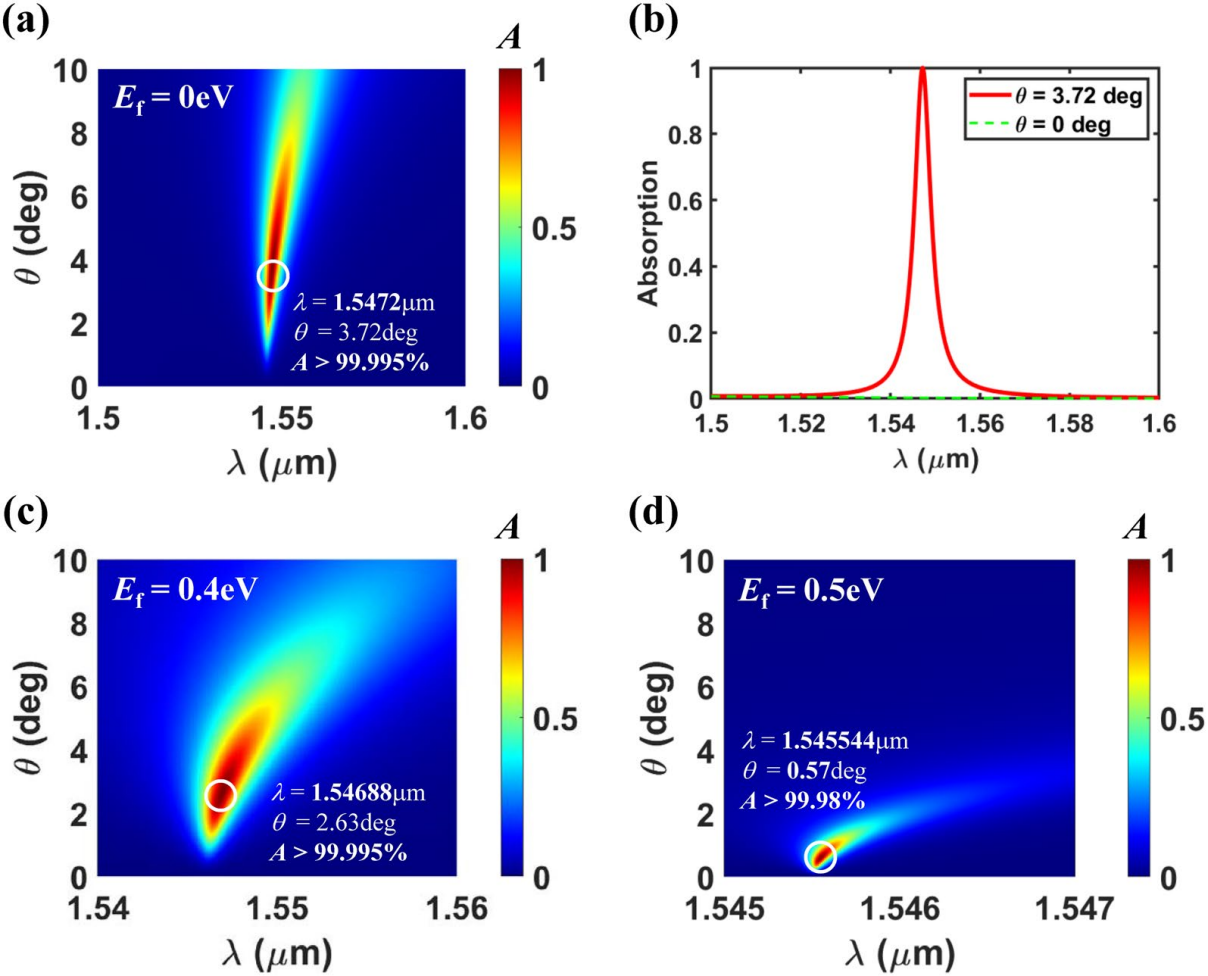
(**a**) Absorption spectra as a function of $\theta$ for the proposed graphene absorber, where *E*_f_ = 0 eV, *Period* = 0.85 $\upmu$m, *FF* = 0.55, *d*_Grat_ = 0.367 $\upmu$m, and *d*_Slab_ = 0.052 m$\mu$. (**b**) Absorption spectra extracted at two incidence angles ($\theta$ = 0 and 3.72 deg), which are extracted from (**a**). Absorption spectra as a function of $\theta$ when (**c**) *E*_f_ = 0.4 eV and (**d**) *E*_f_ = 0.5 eV, assuming that all the remaining parameters are same as those of (**a**).

The performance of the designed graphene perfect absorber is numerically demonstrated, especially focused on the loss variation adaptive *Q*-factor control via a proper choice of the incidence angle. All numerical calculation in this work has been conducted with the RCWA simulation^37^. Figure 2a shows the calculated absorption spectra as a function of $\theta$ for the designed perfect absorber with undoped graphene (*E*_f_ = 0). A strong absorption peak branch is observed for ~ 2 degree < $\theta$  <  ~ 6 degree, and perfect absorption (*A* > 99.995%) occurs at $\theta$ = 3.72 degree and $\lambda$ = 1.5472$\upmu$m as marked by the white open circle. The enhanced absorption is attributed to the quasi-BIC mode, which will be confirmed later in Fig. 5b. The absorption spectrum for the optimal angle ($\theta$ = 3.72 degree) is compared to that for the normal incidence case in Fig. 2b. For the normal incidence, only a GMR mode is excited since the excitation of the symmetry-protected (ideal) BIC mode is forbidden. So, the small absorption (*A* <  < 1%) for the normal incidence implies that the absorption due to the GMR mode is negligible and the one-port system mimicking concept really works. The detailed analysis on the operation principle of the designed device will be discussed in the next section.

The excellent feature of the designed perfect absorber is that loss adaptive perfect absorption is achievable via a proper choice of the incidence angle. Especially, perfect absorption can be achieved even if the loss (or the absorption coefficient) of the absorbing material is extremely low. This is attributed to the unique property of the quasi-BIC with an extremely large *Q*-factor (a vanishingly small leakage rate) as the incident angle approaches zero. So, if the absorption coefficient of the absorbing material becomes smaller, the critical coupling condition, that is, the balance between the leakage rate and the loss rates can be satisfied at a smaller incident angle. To demonstrate this, we investigated the angle dependent absorption performance of the designed device for two different doping levels (or Fermi levels, i.e. *E*_f_) of graphene, which is plotted in Fig. 2c,d. The loss of graphene rapidly decreases with a Fermi level increase as seen in Fig. 1b. For *E*_f_ = 0.5 eV and mobility *Mo* = 0.5 m^2^/Vs, the loss rate of graphene corresponds to ~ 1/40 of the undoped case of the same mobility at $\lambda$ ~ 1.55 $\upmu$m. Despite of the significant loss rate change, perfect absorption is still achieved at a lower incidence angle. The bandwidth of absorption spectrum decreases as *E*_f_ increases, which is due to the increased *Q*-factor of the quasi-BIC mode at the lower incidence angle. It is straightforward that perfect absorption can also be achieved for the increased loss of graphene at the larger incidence angle, which is not shown here. Therefore, even for the unwanted loss variation of graphene, the designed device can achieve perfect absorption via adaptive control of the incidence angle.

### Quasi-BICs in a SWG

**Figure 3:**
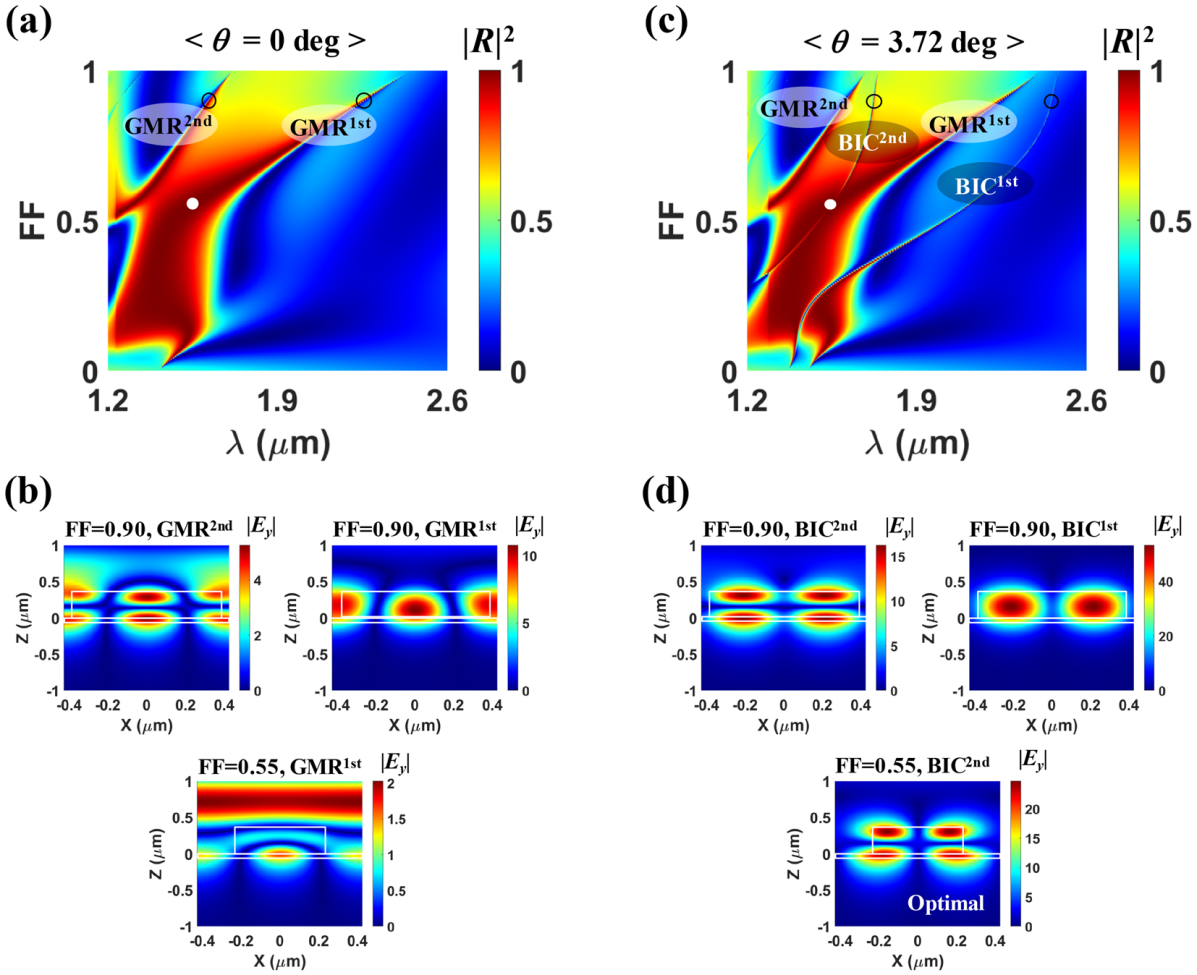
Reflectance spectra as a function of *FF* at (**a**) $\theta$ = 0 (normal incidence) and (**c**) $\theta$ = 3.72 deg (oblique incidence) for the proposed structure without graphene, where *Period* = 0.85 $\upmu$m, *d*_Grat_ = 0.367 $\upmu$m, and *d*_Slab_ = 0.052 $\upmu$m. (**b**) and (**d**) indicate the normalized electric field distributions (|*E*_*y*_|) at different conditions marked by open and solid circles in (**a**) and (**c**) respectively are plotted: *FF* = 0.90 (upper panel), *FF* = 0.55 (lower panel).

In order to analyze the operation principle of the proposed perfect absorber, we first investigate the reflection properties when the absorbing medium is removed from the designed absorber. Figure 3a shows the fill factor (*FF*) dependence of reflectance (|*R*|^2^) for normal incidence ($\theta$= 0), where only the GMR modes are excited. Note that no BIC mode is excited due to the lateral anti-symmetry. When *FF* is very large or small, the reflectance branches become very narrow, corresponding to sharp spectral resonances, due to small leakage rates of the GMRs in the SWG that are noted as GMR^1st^ and GMR^2nd^^40,41^. The field profiles of those modes at *FF* = 0.90 are plotted in the upper panels of Fig. 3b, indicating their origins. In those plots, the field of the incident/reflected plane wave in the air is not clearly observable because of the strong field enhancement in the SWG caused by their high *Q*-factor. For moderate fill factors (~ 0.2 < *FF* <  ~ 0.6), the *Q*-factor of GMR^1st^ considerably decreases and thus, it forms a broadband reflector in conjunction with the F-P like background scattering^20,42^. As plotted in the lower panel of Fig. 3b, the field profile of the low-Q GMR^1st^ near the reflection peak condition (*FF* = 0.55, $\lambda$ = 1.5472 $\upmu$m, $\theta$ = 0, which is marked by the white solid circle in Fig. 3a) shows relatively weak field amplitude in the SWG, which is significantly modified compared to the case of *FF* = 0.90 owing to the strong scattering strength of the ridge part. However, the original guided-mode property is still observed. One important feature to note here is that most of the field is confined in the slab region of the SWG, so that GMR^1st^ mode will experience almost no loss when monolayer graphene is placed on top of the SWG.

For oblique incidence ($\theta$ = 3.72 degree), two high-Q branches additionally appear on the reflectance map (Fig. 3c). They correspond to the first- and the second-order quasi-BIC modes noted as BIC^1st^ and BIC^2nd^ and cross different regions of the broad reflection branch due to the GMR mode. These quasi-BIC modes are manifested by breaking the symmetry of ‘symmetry-protected BICs’ with infinite *Q*-factor^24–28^. Note that for the symmetry-protected BICs, leakage radiation to the surface normal direction is forbidden because of symmetry incompatibility with the external radiation, as checked in Fig. 3a. The symmetry-protection of the BIC originates from the destructive interaction of two leaky guided mode propagating in the opposite direction. So, for the oblique incidence, the imbalanced excitation of the two destructive leaky guided modes forms the imperfect (quasi) BIC which can be coupled to the external plane wave. The field profiles of BIC^1st^ and BIC^2nd^ at *FF* = 0.90 are plotted in the upper panels of Fig. 3d, where the vertical field distributions confirm that the quasi-BICs originates from the leaky guided modes. The quasi-BIC mode relevant to perfect absorption in our designed device is BIC^2nd^, which will be discussed later. As shown in the lower panel of Fig. 3d, at the optimal condition (*FF* = 0.55, $\lambda$ = 1.5472 $\upmu$m, $\theta$ = 3.72 degree, which is marked by the white solid circle in Fig. 3c), the electric field profile is not perfectly but close to anti-symmetric in the lateral (*x*) direction with respect to the center of the ridge since the contribution of BIC^2nd^ is dominant. Although GMR^1st^ mode is also excited in this case, the energy stored in it is negligible compared to BIC^2nd^ due to the huge *Q*-factor difference. So, much stronger field enhancement is attributed to BIC^2nd^ as well.

### Virtual one-port feature in the interaction between the GMR and the quasi-BIC modes and its angle dependence

**Figure 4:**
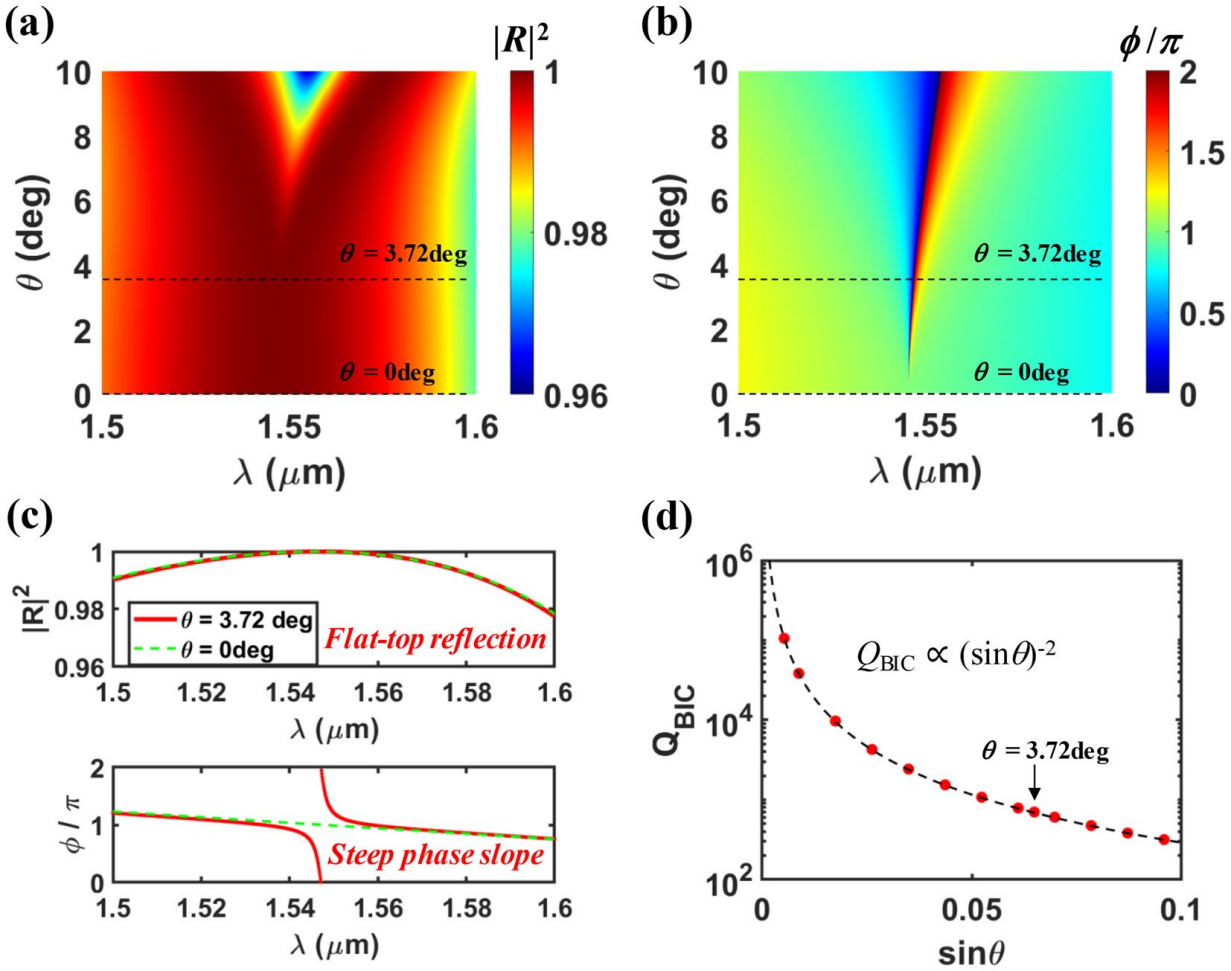
(**a**) Reflectance spectra and (**b**) reflection phase a function of $\theta$ for the proposed structure without graphene, assuming that all the remaining parameters are same as the optimal condition in Fig. 2 (that is, *Period* = 0.85 $\upmu$m, *FF* = 0.55, *d*_Grat_ = 0.367 $\upmu$m, and *d*_Slab_ = 0.052 $\upmu$m). (**c**) Reflectance spectra and reflection phase at two angles ($\theta$ = 0 and 3.72 deg), which are extracted from (**a**) and (**b**) respectively. (**d**) *Q*-factor of quasi-BIC (*Q*_BIC_) as a function of $\theta$, which is extracted by applying CMT model: red solid circle is for CMT model, and black dashed curve is for a proper inverse quadratic equation (*Q*_BIC_ = 2.9∙(sin $\theta$)^-2^) as approximate expression.

Now, we closely investigate the interaction between the GMR and the quasi-BIC modes, and the angle dependence of BIC^2nd^ for the designed structure without graphene layer. Figure 4a,b show reflectance (|*R*|^2^) and reflection phase ($\phi$) spectra as a function of the incidence angle over the wavelength range of the high reflection due to GMR^1st^ mode at normal incidence (~ 1.5 $\upmu$m < $\lambda$  <  ~ 1.6 $\upmu$m). The broadband flat-top reflection close to 100% is maintained up to $\theta$ ~ 5 degree, and as $\theta$ increases further, a reflection dip develops, reaching |*R*|^2^ ~ 96% for $\theta$ = 10 degree. On the other hand, the phase shows steep variation near the wavelength of $\lambda$ ~ 1.55 $\mu$ m for $\theta$ > 0 and the wavelength of $\phi$ = 0 shows slow red shift with increasing $\theta$. From the fact that no steep phase variation is observed at normal incidence ($\theta$= 0), in which the excitation of the BIC is forbidden, we can see that the steep phase variation is due to the resonant excitation of the quasi-BIC (BIC^2nd^), and the wavelength of $\phi$ = 0 corresponds to the resonance wavelength of the quasi-BIC (BIC^2nd^). We can see that the locus of the resonance wavelength of BIC^2nd^ is the same as the strong absorption peak branch in Fig. 2a, which is a clear evidence that BIC^2nd^ is responsible for the absorption in our designed device.

For a clear comparison, two reflectance (reflection phase) spectra for $\theta$ = 0 and 3.72 degree are plotted together in the upper (lower) panel in Fig. 4c. This reveals that in our designed device, the introduction of the high-*Q* BIC^2nd^ does not ruin the broadband flat-top reflection due to the GMR^1st^—the background scattering interaction. It is noteworthy that this kind of interaction between two resonant modes in a single resonator is an unusual phenomenon, which requires triple degeneracy among the two resonant modes and the F-P like background scattering and a proper indirect mutual coupling strength between the two modes as theoretically proved in our previous works^20,43^. Unless these conditions are satisfied, which is more usual, a sharp reflection fluctuation known as Fano line shape appears^44^. Previously, quasi-BIC related Fano resonances are utilized for a narrowband transmission filter at slightly off-normal incidence angle in a 1-D high contrast grating (HGC)^28^ and a narrowband reflection filter in an asymmetric metasurface^29^. As seen in Fig. 3c, even in our designed structure, a sharp reflection dip of *R* ~ 0 is observed when BIC^1st^ interacts with the GMR mode at *FF* = 0.2 and $\lambda$ = 1.4465 $\mu$ m with all other parameters kept the same as the optimal design. The enlarged spectrum plot at *FF* = 0.2 (Fig. S1) and its detailed description is provided in Supplementary information. The interaction between two resonant modes in a two-port asymmetric resonator like the SWG can be modeled as an indirect coupling via the partial reflections at the two port interfaces in the CMT^20,43^. From this CMT modeling, we can find the specific indirect coupling condition between the two degenerate resonant modes for perfect reflection or transmission at the resonance. (Supplementary information) This implies that depending on the indirect mutual coupling condition between the quasi-BIC and the GMR modes, perfect transmission as well as perfect reflection can occur at the resonance. Since the indirect coupling stems from the partial reflections at the surfaces of the SWG, the coupling condition is strongly dependent on *FF*. In our optimal design, the proper coupling condition between BIC^2nd^ and GMR^1st^ modes for perfect reflection is satisfied at *FF* = 0.55, $\lambda$ = 1.5472 $\upmu$m, and $\theta$ = 3.72 degree, while perfect transmission condition happen to be satisfied between BIC^1st^ and GMR^1st^ modes at *FF* = 0.2 and $\lambda$ = 1.4465 $\upmu$m. Since the one-port resonant system mimicking concept requires perfect reflection at resonance, perfect absorption in our designed device is based on BIC^2nd^ and GMR^1st^ modes.

In our present design, the proper coupling condition between BIC^2nd^ and GMR^1st^ modes appears to remain roughly up to $\theta$ ~ 5 degree as seen in Fig. 4a. For a larger angle, the proper coupling condition becomes to break due to the unacceptably reduced *Q*-factor of BIC^2nd^ mode, resulting in the reflectance dip development. Note that the indirect coupling strength is determined by the decay rates of the two modes as well as the reflections at the surfaces of the SWG^20,43^. So, the operation range of $\theta$ for the variable *Q*-factor should be limited. However, this is not the fundamental limit. Our present design is for undoped graphene of a certain condition (Fermi velocity of 10^6^ m/s, and mobility of 0.5 m^2^/Vs). For graphene of a larger loss due to the difference material quality, the device can be redesigned to have a larger optimal incidence angle, and then, the operation range of $\theta$ can be increased.

It is also noteworthy that the reflection characteristics of our designed structure without graphene is similar to the phase shifter based on an ideal asymmetric Fabry–Perot cavity with a perfectly reflecting bottom mirror^38,45,46^. Therefore, it can be stated that the designed structure behaves like a virtual one-port resonant system, mimicking the system composed of a single-mode resonator and an external mirror. In our proposed perfect absorber structure, BIC^2nd^ works as a lossy mode while GMR^1st^ works as an internal mirror in conjunction with the background scattering as aforementioned.

Another key feature of the proposed structure is that the slope of the reflection phase at the resonance, which is directly related to *Q*-factor of BIC^2nd^, shows $\theta$ dependence as seen in Fig. 4b. The steeper phase slope implies the higher *Q*-factor (or lower leakage rate); the smaller incidence angle, the higher *Q*-factor. This is because a smaller incidence angle induces a weaker structural asymmetry and consequently, a less distortion of the symmetry-protected BIC. For the quantitative analysis of the angle dependent *Q*-factor of BIC^2nd^, we extracted the leakage rate of BIC^2nd^ by fitting the CMT model to the reflection phase spectrum calculated with the RCWA. According to the CMT, the reflection phase in a lossless one-port system with a single resonance is given by

1 $$\phi (\omega ) = 2\tan^{ - 1} \left( {\frac{{\omega - \omega_{o} }}{{\gamma_{leak} }}} \right), $$

where $\omega$_o_ and *γ*_leak_ are a resonant frequency and a leakage (or external decay) rate, respectively^5,38^. The *Q*-factor of BIC^2nd^ is calculated by *Q*_BIC_ = $\omega$_o_/2*γ*_leak_, which is plotted in Fig. 4d. In particular, *Q*_BIC_ ~ 702 at the optimal angle of $\theta$ = 3.72 degree. As expected, *Q*_BIC_ increases toward infinity as $\theta$ approaches zero. For a small incident angle, *Q*_BIC_ is inversely proportional to the square of the asymmetry parameter (sin $\theta$), similar to the result reported in ref.^29^ in which asymmetry was introduced by the rotation angle of the individual meta-atom in the asymmetric metasurface for a narrowband reflection filter.

### Optimal incidence angle selection for loss adaptive perfect absorption

The optimal incidence angle selection for perfect absorption satisfying the critical coupling condition is analyzed with the CMT. According to the CMT, absorption efficiency in a lossy one-port system with a single resonance is given by

2 $$ A(\omega ) = \frac{{4\gamma_{leak} \gamma_{loss} }}{{(\omega - \omega_{o} )^{2} + (\gamma_{leak} + \gamma_{loss} )^{2} }}, $$

where *γ*_leak_ and *γ*_loss_ and are the leakage and the loss (or the internal decay) rates, respectively^5,6^. Perfect absorption can be reached when the critical coupling condition (*γ*_leak_ = *γ*_loss_) is satisfied at the resonance ($\omega$ = $\omega$_o_).

**Figure 5:**
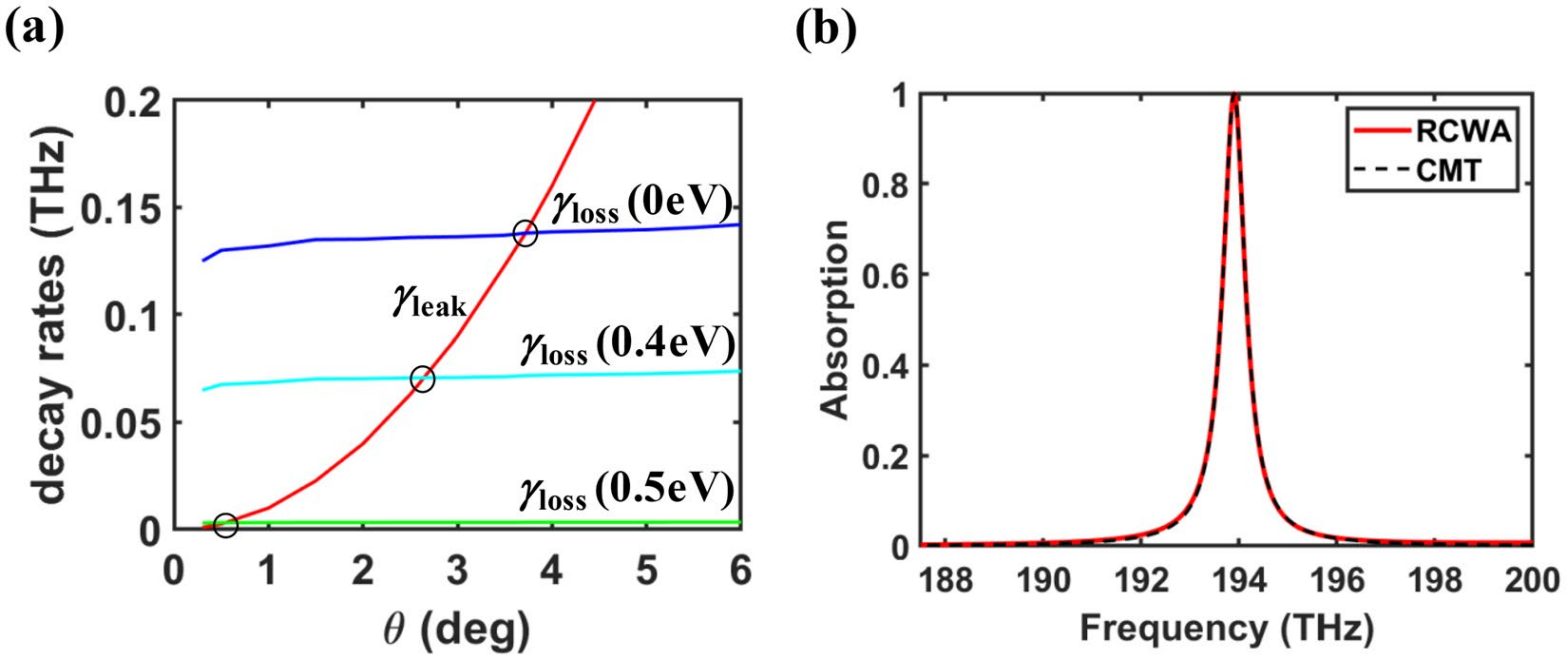
(a) Leakage and loss rates as a function of $\theta$ for different Fermi-levels of *E*_f_ = 0, 0.4, and 0.5 eV, which are obtained by applying CMT model. Three open circles indicate the critical coupling condition for each *E*_f_. (b) Comparison of CMT and the RCWA calculation results for *E*_f_ = 0 eV. All the remaining parameters are same as those of Fig. 2.

Once *γ*_leak_ is obtained via fitting the CMT model (1) to the reflection phase spectrum calculated with the RCWA as mentioned in the previous section, we can also extract *γ*_loss_ by fitting the CMT model (2) to the absorption spectra (Fig. 2) calculated with the RCWA, which are plotted as a function of $\theta$ in Fig. 5a. As $\theta$ increases, *γ*_leak_ increases rapidly, while *γ*_loss_ is almost constant, which is consistent with the physical reasoning. From this, it is straightforward that the optimal incidence angle will be changed if the loss rate of the absorbing material varies. In our designed absorber with undoped graphene (*E*_f_ = 0), it is confirmed that the critical coupling occurs at $\theta$ = 3.72 degree, which is the optimal incidence angle for perfect absorption. For the doped graphene cases of *E*_f_ = 0.4 eV and 0.5 eV, the optimal incidence angles for the critical couplings are $\theta$ = 2.63 degree and 0.57 degree, respectively. In Fig. 5b, the numerically calculated absorption spectra and the CMT model are compared for the designed absorber with undoped graphene at the optimal incidence angle, where we can see the excellent agreement between them. This confirms again that the designed absorber behaves like a lossy one-port resonant system in the vicinity of the BIC^2nd^ resonance.

## Conclusion

In conclusion, we have numerically demonstrated a graphene perfect absorber with a function of adaptive control of *Q*-factor for loss variation, which is based on the quasi-BIC and the GMR modes in a SWG. In the device design, the one-port resonant system mimicking concept was adopted. The device structure is quite simple and easy to fabricate; no external mirror is required, and monolayer graphene is placed on the ridge of the SWG. The proposed perfect absorber scheme can be used for any kind of ultrathin absorbing media including various 2-D materials.

## Methods

To numerically investigate and analyze the reflection and absorption properties in the proposed perfect absorber, we used two-dimensional RCWA (a commercial software, DiffractMOD)^37^ and the coupled mode theory (CMT) fitting for a lossy and lossless one-port resonant system^5,6,38^. In the RCWA simulation, more than 300 harmonics were applied to guarantee accuracy around the resonant wavelength. In all our calculations, the complex permittivity of graphene ($\varepsilon$_*g*_) was calculated using Kubo formulation based on the local random phase approximation for various *E*_f_^19,39^, assuming graphene thickness of 0.34 nm, Fermi velocity of 10^6^ m/s, and mobility of 0.5 m^2^/Vs.

## Supplementary Information

Supplementary Information.
