# Supplementary material for: Graphene perfect absorber with loss adaptive Q-factor control function enabled by quasi-bound states in the continuum
Source: Sci Rep. 2021 Nov 24;11:22819. doi: 10.1038/s41598-021-02318-8 (PMC8613217; doi:10.1038/s41598-021-02318-8)
Supplement: Supplementary file 1 — Supplementary Information. [file 41598_2021_2318_MOESM1_ESM.pdf]

Supplementary Information

Graphene perfect absorber with loss adaptive *Q*-factor control function enabled by quasi-bound states in the continuum

Sangjun Lee, Joohyung Song, and Sangin Kim*

Department of Electrical and Computer Engineering, Ajou University, Suwon 16499, Korea

Email: sangin@ajou.ac.kr

Section 1. Band-pass filtering based on quasi-BIC

In the main text, we have focused on only BIC^{2nd} as a desirable resonance mode for obtaining perfect absorption. Here, we discuss the other quasi-BIC mode (that is, BIC^{1st}) which provides not flat-top reflectance but steep reflectance dip around the resonance, as plotted in Fig. S1. This property is very similar to narrowband transmission (or band-pass) filtering in ref. [S1]. The band-pass filtering is attributed to highly directional leakage radiation of BIC^{1st} to the bottom of SWG through another type of indirect coupling condition between BIC^{1st} and GMR^{1st}. Finally, the absorber based on BIC^{1st} cannot be treated as one-port resonant system, and thus it is impossible to obtain perfect absorption, unlike BIC^{2nd} case. As will be discussed in the next section, from the CMT modeling, we can find that depending on the indirect mutual coupling condition between the quasi-BIC and the GMR modes, perfect transmission as well as perfect reflection can occur at the resonance.

To compare the characteristics of two quasi-BICs, it is meaningful to study carefully the electric field profiles. For optimal *FF* of 0.55, BIC^{2nd} (see the lower panel of Fig. 3(d) in main text) and low-Q GMR^{1st} (see the lower panel of Fig. 3(b) in main text) have the field configurations of vertically odd modes across slab and ridge parts in SWG structure, that is, the electric fields are concentrated on each region of slab and ridge parts. Thus, we can classify both of BIC^{2nd} and low-Q GMR^{1st} as vertically odd modes, despite of their opposite symmetry with respect to *y-z* plane. On the other hand, as plotted in Fig. S1, for $FF = 0.2$, the field profile of BIC^{1st} is completely different from GMR^{1st}, considering that the strong fields in BIC^{1st} are concentrated on not ridge but slab part. That is, BIC^{1st} corresponds to vertically even mode, whereas low-Q GMR^{1st} corresponds to vertically odd mode. We surmise that the similarity in electric fields between quasi-BIC and GMR^{1st} is closely bound up with a directional leakage radiation of quasi-BICs, which seems to be a rich topic of our future research.

Similar to incident angle dependence of *Q*-factor in BIC^{2nd}, the band-pass filter based on BIC^{1st} also has the higher *Q*-factor (or lower bandwidth) as incident angle decreases, as shown in Fig. S2. This means that a weaker oblique incidence induces a less distortion of (ideal) symmetry-protected BIC due to a weaker structural asymmetry. For normal incidence, any features in the reflectance disappear because symmetry-protected BIC with infinite *Q*-factor cannot be excited by incident light.

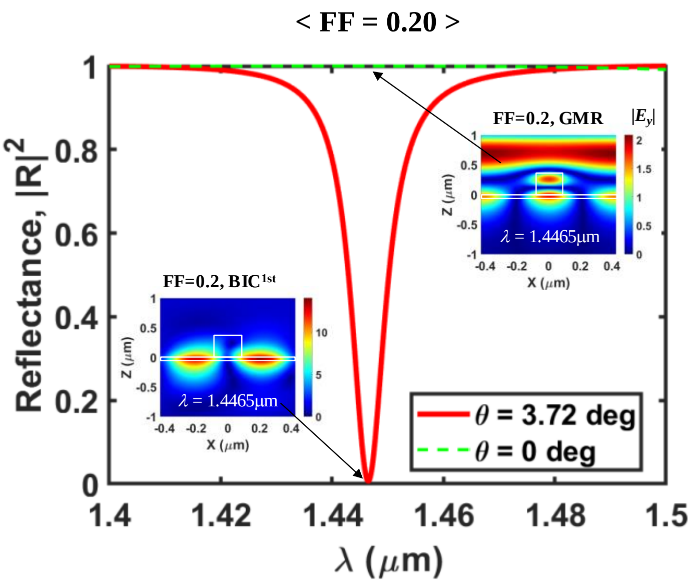

Figure S1. Reflectance spectra at $\theta = 0$ (dashed green) and 3.72 deg (solid red) for the proposed structure without graphene, where all the remaining parameters are same as the optimal condition in Fig. 3 excepting that FF = 0.2 (that is, *Period* = 0.85 μm, FF = 0.20, d_{Grat} = 0.367 μm, and d_{Slab} = 0.052 μm) The insets indicate normalized electric field distributions ($|E_y|$) at λ = 1.4465 μm.

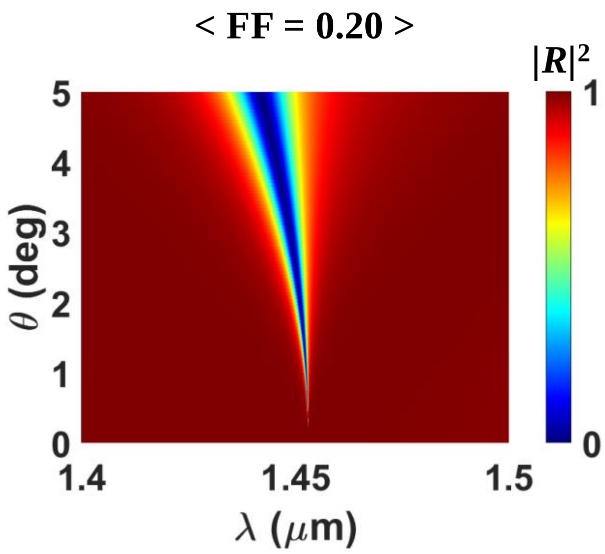

Figure S2. Reflectance spectra a function of θ for the proposed structure without graphene, assuming that all the remaining parameters are same as those in Fig. S1. (that is, *Period* = 0.85 μm, FF = 0.20, d_{Grat} = 0.367 μm, and d_{Slab} = 0.052 μm)

Section 2. Transmission or reflection characteristics due to the interaction between two resonant modes in an asymmetric resonator

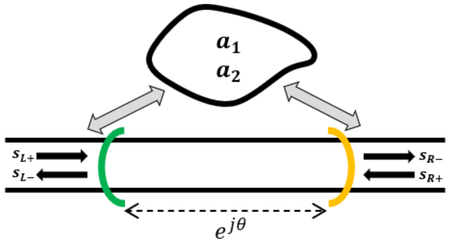

Figure S3. Schematic model of a two-port system describes an asymmetric resonator supported by two resonant modes and an internal wave propagating channel with partial reflections

Using the temporal coupled-mode theory, the behavior of an asymmetric two-port system supporting two resonant modes is described by rate equations [S2]:

$$\frac{da_1}{dt} = \left(j\left(\omega_1 - j\frac{\gamma_1-\gamma_1^*}{2}\right) - \frac{\gamma_1+\gamma_1^*}{2}\right)a_1 - \mu a_2 + \sqrt{\frac{\gamma_1+\gamma_1^*}{2}} \cdot e^{j\varphi_{L1}} \cdot s_{L+} + \sqrt{\frac{\gamma_1+\gamma_1^*}{2}} \cdot e^{j\varphi_{R1}} \cdot s_{R+}, \tag{1}$$

$$\frac{da_2}{dt} = \left(j\left(\omega_2 - j\frac{\gamma_2-\gamma_2^*}{2}\right) - \frac{\gamma_2+\gamma_2^*}{2}\right)a_2 - \mu a_1 + \sqrt{\frac{\gamma_2+\gamma_2^*}{2}} \cdot e^{j\varphi_{L2}} \cdot s_{L+} + \sqrt{\frac{\gamma_2+\gamma_2^*}{2}} \cdot e^{j\varphi_{R2}} \cdot s_{R+}, \tag{2}$$

where a_i is the normalized amplitude of the i-th mode, ω_i is the resonant frequency of the i-th mode when the intermodal interaction effect is excluded, γ_i is the decay coefficient of the i-th mode, μ is the mutual coupling coefficient between the two modes, $\varphi_{Li/Ri}$ is the phase of coupling coefficient of i-th mode from L/R-port, and $s_{L+(-)/R+(-)}$ is the amplitude of incoming (outgoing) wave at L/R-port. It is assumed that the two modes interact with each other indirectly via the Fabry-Perot (F-P) like background scattering stemming from the partial reflections in the internal wave propagating channel. So, both γ_i and μ are complex value [S2]. It is also assumed that reflection coefficient of the background scattering is zero.

For single-sided illumination from L-port $(s_{L+} \neq 0, s_{R+} = 0)$, frequency dependent transmission and reflection coefficients of the system are given by

$$T = \frac{s_{R-}}{s_{L+}} = e^{j\left(\theta+\frac{\pi}{2}\right)} \cdot \left(1 - \frac{-\mu \cdot \sqrt{\frac{\gamma_1+\gamma_1^*}{2} \cdot \frac{\gamma_2+\gamma_2^*}{2}} \cdot \left(e^{j(\varphi_{R1}-\varphi_{R2})} + e^{-j(\varphi_{R1}-\varphi_{R2})}\right) + \left(j\left(\omega - \left(\omega_1 + j\frac{\gamma_1-\gamma_1^*}{2}\right)\right) + \frac{\gamma_1+\gamma_1^*}{2}\right) \cdot \frac{\gamma_2+\gamma_2^*}{2} + \left(j\left(\omega - \left(\omega_2 + j\frac{\gamma_2-\gamma_2^*}{2}\right)\right) + \frac{\gamma_2+\gamma_2^*}{2}\right) \cdot \frac{\gamma_1+\gamma_1^*}{2}}{\left(j\left(\omega - \left(\omega_1 + j\frac{\gamma_1-\gamma_1^*}{2}\right)\right) + \frac{\gamma_1+\gamma_1^*}{2}\right) \cdot \left(j\left(\omega - \left(\omega_2 + j\frac{\gamma_2-\gamma_2^*}{2}\right)\right) + \frac{\gamma_2+\gamma_2^*}{2}\right) - \mu^2}\right) \tag{3}$$

and

$$R = \frac{s_{L-}}{s_{L+}} = \frac{-2\mu \cdot \sqrt{\frac{\gamma_1+\gamma_1^*}{2} \cdot \frac{\gamma_2+\gamma_2^*}{2}} \cdot e^{j(\varphi_{R1}+\varphi_{R2})} + \left(j\left(\omega - \left(\omega_1 + j\frac{\gamma_1-\gamma_1^*}{2}\right)\right) + \frac{\gamma_1+\gamma_1^*}{2}\right) \cdot \frac{\gamma_2+\gamma_2^*}{2} \cdot e^{j2\varphi_{R2}} + \left(j\left(\omega - \left(\omega_2 + j\frac{\gamma_2-\gamma_2^*}{2}\right)\right) + \frac{\gamma_2+\gamma_2^*}{2}\right) \cdot \frac{\gamma_1+\gamma_1^*}{2} \cdot e^{j2\varphi_{R1}}}{\left(j\left(\omega - \left(\omega_1 + j\frac{\gamma_1-\gamma_1^*}{2}\right)\right) + \frac{\gamma_1+\gamma_1^*}{2}\right) \cdot \left(j\left(\omega - \left(\omega_2 + j\frac{\gamma_2-\gamma_2^*}{2}\right)\right) + \frac{\gamma_2+\gamma_2^*}{2}\right) - \mu^2}. \tag{4}$$

When the two resonant modes are degenerated with the intermodal interaction included, the resonance frequency of the system will be $\omega_o = \omega_1 + j\frac{\gamma_1-\gamma_1^*}{2} = \omega_2 + j\frac{\gamma_2-\gamma_2^*}{2}$. From (3), zero transmission at the resonance frequency $(T(\omega_o) = 0)$ requires

$$\mu = \sqrt{\frac{\gamma_1+\gamma_1^*}{2} \cdot \frac{\gamma_2+\gamma_2^*}{2}} \cdot e^{j(\varphi_{R1}-\varphi_{R2})}. \tag{5}$$

From (4), the conditions for zero reflection at the resonance frequency $(R(\omega_o) = 0)$ can also be found as

$$\mu = \sqrt{\frac{\gamma_1+\gamma_1^*}{2} \cdot \frac{\gamma_2+\gamma_2^*}{2}} \cdot \cos(\varphi_{R1} - \varphi_{R2}) \text{ and } \varphi_{R1} - \varphi_{R2} \neq m\pi. \text{ (m is zero or an integer)} \tag{6}$$

Note that the condition of $\varphi_{R1} - \varphi_{R2} \neq m\pi$ is required to avoid the zero-denominator case in (4). $\varphi_{R1} - \varphi_{R2} = m\pi$ results in the case of zero divided by zero, corresponding to $R(\omega_o) = 1$, that is, $(T(\omega_o) = 0)$.

Therefore, in the asymmetric two-port single resonator supporting two degenerate modes, both perfect reflection and perfect transmission at the resonance can happen depending on the mutual coupling coefficient, which is determined by the partial reflections in the wave propagation channel and the decay rates of the resonant modes inside the channel [S2].

REFERENCES

[S1]. Foley, J. M., Young, S. M. & Phillips, J. D. Narrowband mid-infrared transmission filtering of a single layer dielectric grating. *Appl. Phys. Lett.* **103**, 071107 (2013).

[S2]. Song, J., Heo, H., Lee, S. & Kim, S. Mirror-less unidirectional radiation in an asymmetric single resonator. Preprint at https://doi.org/10.36227/techrxiv.15020034.v1 (2021).
